# Hedgehog Zoonoses

**DOI:** 10.3201/eid1101.040752

**Published:** 2005-01

**Authors:** Patricia Y. Riley, Bruno B. Chomel

**Affiliations:** * University of California, Davis, Davis, California, USA

**Keywords:** hedgehogs, zoonoses, Salmonella, ringworm, Mycobacterium marinum, synopsis

## Abstract

Exotic pets, including hedgehogs, have become popular in recent years among pet owners, especially in North America. Such animals can carry and introduce zoonotic agents, a fact well illustrated by the recent outbreak of monkeypox in pet prairie dogs. We reviewed known and potential zoonotic diseases that could be carried and transmitted by pet hedgehogs or when rescuing and caring for wild-caught hedgehogs.

## Abstract 2

Pet hedgehogs pose a zoonotic risk to owners.

Pets play an important role in societies throughout the world (*1*). They are important companions in many households, contributing to the physical, social and emotional development of children and the well-being of their owners, especially the elderly (*1*). Although pets offer significant benefits, potential hazards are associated with pet ownership (*1*). Exotic animals are increasingly being invited into homes as pets (*2*). However, neither pet owners nor nonveterinary healthcare providers are sufficiently knowledgeable about the potential of many of these animals to transmit zoonotic diseases (*2*).

Hedgehogs are small, nocturnal, spiny-coated insectivores that have been gaining popularity as exotic pets (*3*). These animals are considered to be unique, low-maintenance pets (*4*), and an estimated 40,000 households in the United States now own them (*5*). These animals originally arrived from Europe, Asia, and Africa, and although several species exist, 2 in particular are commonly seen as pets (*3*): the European hedgehog, *Erinaceus europaeus*, and the smaller African pygmy hedgehog, *Atelerix albiventris* (*3*). The importation of these pets from Africa to the United States has been prohibited since 1991 (Title 9 Code of Federal Regulations Section 93.701) due to their potential to carry foot-and-mouth disease, a foreign animal disease of serious economic concern to the livestock industry (*6*). In the United States, persons who sell hedgehogs are required to have a U.S. Department of Agriculture (USDA) license (http://www.nal.usda.gov/awic/newsletters/v9n1/9n1aphis.htm). In some states, such as Arizona, California (http://www.dfg.ca.gov/licensing/pdffiles/fg1518.pdf), Georgia, Hawaii, Maine, Pennsylvania, Vermont, and Washington D.C., owning a hedgehog as a pet is illegal, (www.hedgehogwelfare.org), as is the case in some of New York City boroughs (Manhattan, Brooklyn, Queens, Bronx, Staten Island) (source: http://www.petfinder.org/shelters/CT171.html).

Hedgehogs live in a variety of habitats where they dig their own burrows, spend most of the daylight hours asleep, and emerge at night to forage (*3*). Hedgehogs are characterized by short, grooved spines covering the entire dorsum of the body (*3*). When frightened by an unfamiliar sound or movement, the animal rolls into a tight ball (*3*). In this defensive posture, the hedgehog brings its snout and limbs close under its body, causing the spines to become erect (*3*). The spines, modified hairs having a spongy matrix and outer keratinous shaft (*7*), are not barbed (*8*). The spines rarely cause serious injury to handlers (*8*) but can readily penetrate the skin (*7*). However, 1 report described 3 patients in whom an acute, transient, urticarial reaction developed after contact with the extended spines of pet hedgehogs (*7*)

Hedgehogs display an unusual behavior called “anting” or “anointing” (*7*). When first encountering a new or interesting object or food, the animal will lick the substance repeatedly until a frothy saliva forms in its mouth (*3*). The animal then rubs the excess saliva and froth onto its skin and spines (*3*). This behavior may cause saliva to accumulate on the spines, making the hedgehog less palatable to predators (*7*).

In addition to the contact urticaria that has been reported in some hedgehog handlers (*7*) hedgehogs pose a risk for a number of potential zoonotic diseases (*2*). Major microbial infections associated with hedgehogs include bacteria such as *Salmonella* and *Mycobacteria*, as well as some fungal and viral diseases (*2*). Many disease conditions can cause immunodeficiency in humans; the most notable is AIDS (*9*). Similarly, immunosuppressive strategies employed to prevent rejection of bone marrow or solid organ transplants render such patients extremely susceptible to viral and mycobacterial infections (*9*). An increasing percentage of the population is becoming susceptible to severe diseases associated with exotic pet ownership, as illustrated by the recent monkeypox outbreak in pet prairie dogs (*10*). Immunocompromised persons may be at increased risk for infections from hedgehogs and should be particularly careful.

The following review is focusing on the zoonotic or potentially zoonotic agents carried by hedgehogs (Table). The risks are particularly of concern for people rescuing wild-caught hedgehogs and adopting them as pets. We distinguished major established zoonotic infections, such as salmonellosis or ringworm, from other less common or potential zoonoses carried by hedgehogs.

**Table:** Zoonotic and potentially zoonotic viral, bacterial, protozoal, and mycotic zoonoses of hedgehogs

	Bacterial	Viral	Protozoal	Mycotic
I. Confirmed zoonotic diseases carried by hedgehogs:	*Salmonella* spp.* *Yersinia pseudotuberculosis†* *Mycobacterium marinum*	Rabies Herpesvirus, including human herpes simplex		Trychophyton mentagrophytes var. erinacei* *Microsporum* spp.†
II. Potential zoonotic diseases carried by hedgehogs:	*Chlamydia psittaci* *Coxiella burnetii* *Yersinia pestis*	Arboviruses Tickborne encephalitis Crimean-Congo hemorrhagic fever Tahyna virus Bhanja virus Paramyxovirus	*Cryptosporidium* *Toxoplasma gondii*	*Candida albicans*

*Most common zoonoses. †Common zoonoses.

## Bacterial Zoonoses

Salmonellosis is the main zoonotic disease associated with hedgehogs, as well as several other exotic pet species (*4*). Although affected hedgehogs can display anorexia, diarrhea, and weight loss, ≈28% of hedgehogs are asymptomatic carriers (*11*). Several recent reports have shown that hedgehogs play a major role in the transmission of *Salmonella* Tilene, a rarely encountered serotype of humans (*4*,*6*,*12*). In 1994, *Salmonella* serotype Tilene infection was diagnosed in 10-month-old girl in the state of Washington (*6*). The source of the organism was traced back to African pygmy hedgehogs raised by the child’s family. Although the infant had no direct contact with the hedgehogs, the animals were handled frequently by 1 member of the family. Cultures from the child’s asymptomatic parents were negative, and the breeding herd of 80 hedgehogs was apparently healthy, but a stool sample from 1 of 3 hedgehogs yielded *S.* Tilene. A second *S.* Tilene case was reported later that year from Texas (*6*). That patient’s family also owned a hedgehog.

From 1995 to 1997, 10 laboratory-confirmed cases of *S.* Tilene infection (*12*) were reported in Canada. With 1 exception, all cases occurred in children, with 5 cases in children <3 years. All but 1 patient came from or had contact with families owning African pygmy hedgehogs. The adult and older children cases were either directly responsible for the care of the hedgehogs or had other direct contact with the animals. Four of the cases were associated with breeding herds of hedgehogs.

The risk for such infections can be reduced by hand washing after handling of pets, especially before eating or handling food and by avoiding contact with pets’ feces (*6*). Similarly, pets should be fed, housed, and handled properly, and all pets should be carefully watched for signs of illness and treated appropriately when ill (*12*).

Two human outbreaks of salmonellosis caused by *S.* Typhymurium 4,5,12:I:1,2 were reported in Norway; *Salmonella*-infected hedgehog populations most likely constituted the primary source of human infection, as ≈40% of the animals tested carried the same strain (*13*). Furthermore, the pulsed-field gel electrophoresis profiles of isolates from hedgehogs and human beings were identical within each of the 2 outbreak areas. In an evaluation of mortality of hedgehogs in East Anglia and presence of possible zoonotic diseases, salmonellosis was found to be the most common zoonotic infection (*14*). *S.* Enteritidis PT 11 was found in 13 hedgehogs, which suggests a special association between this phage type of *S.* Enteritidis and hedgehogs in East Anglia. This phage type has also been isolated from human beings. *S.* Typhymurium PT 104 was also isolated from 1 animal in the same study. Overall, the incidence of *Salmonella* was 18.9% of 74 hedgehogs (*14*).

These authors also reported the first isolation of *Yersinia pseudotuberculosis* in the United Kingdom from 2 hedgehogs that died at a rehabilitation center in Berkshire (*14*). The organism primarily causes a gastroenteritis characterized by a self-limited mesenteric lymphadenitis, which mimics appendicitis. Postinfectious complications include erythema nodosum and reactive arthritis.

Another condition of zoonotic concern reported in hedgehogs is systemic mycobacteriosis caused by *Mycobacterium marinum* (*15*). A European hedgehog was brought for treatment with nonsuppurative masses in the subcutis of the ventral cervical region (*15*). The animal worsened and died. On necropsy, multiple granulomatous lesions found in lymph nodes, lungs, spleen, liver, and heart were positive for *M. marinum* in culture. In the reported case, the hedgehog apparently acquired *M. marinum* from the fish tank in which the animal was housed at a pet store (*15*). In humans, the organism is associated with a cutaneous disease called “fish-tank granuloma,” which is frequently contracted from contact with aquariums (*16*). The organism typically gains entry through some wound or abrasion in the skin, such as may be produced by hedgehog spines, and may spread systemically along the lymphatic system. The resulting disease produces lesions resembling those of sporotrichosis, tularemia, nocardiosis, and blastomycosis.

In Madagascar, *Y. pestis* was isolated from an endemic hedgehog (*Hemicentetes nigriceps*), and plague antibodies were detected in another endemic hedgehog species (*Tenrec ecaudatus*) (*17*). However, no human infection directly related to hedgehog exposure has been documented.

In a serosurvey of European hedgehogs in Styria, Austria, antibodies against *Coxiella burnetii* (Q fever), *Chlamydia* (ornithosis), and *Toxoplasma gondii* (toxoplasmosis) were detected among 64 animal tested (*18*). Potential infection of persons who are rehabilitating or caring for rescued hedgehogs by these infectious agents should therefore be considered.

## Mycotic Zoonoses

Dermatophytosis, or ringworm, has been described in the hedgehog (*8*). Lesions in the animal are similar to those found in other species and include nonpruritic, dry, scaly skin with bald patches and spine loss (*8*). *Trichophyton mentagrophytes* var. *erinacei* is the dermatophyte most commonly isolated from the quills and underbelly of hedgehogs, although *Microsporum* spp. have also been reported (*8*). Hedgehogs can be asymptomatic carriers of these fungi (*8*), and herein lies their potential for zoonotic transmission.

Several reports demonstrate the ability of the hedgehog to transmit dermatophytes to humans (*19*–*21*). *Trichophyton mentagrophytes* var. *erinacei* causes an extremely inflammatory and pruritic eruption, which frequently resolves spontaneously 2–3 weeks after onset (*8*). The disease may present as a localized rash with pustules at the edges and a thickened and intensely irritating area in the center of the lesion (*19*).

In 1 report of 3 human ringworm cases, 1 of the patients merely handled the hedgehog in a pet store for 1 to 2 minutes (*21*). A second patient observed multiple annular, erythematous, bullous lesions on her legs, arms, and abdomen within 1 week of purchasing an African pygmy hedgehog from a pet store.

In a second report of human ringworm associated with handling of European hedgehogs, a patient had raised erythematous lesions on her right wrist and 2 fingers of the left hand (*20*). Because the patient was pregnant at the time, she was treated with topical antifungals only. After 1 month, the skin eruption had spread, with resultant destruction of the nails on both affected fingers. When the patient was able to be treated systemically, the infection cleared within 8 weeks.

In addition to the dermatophytes, hedgehogs have been reported to be infected with *Candida albicans* (*22*,*23*). Intestinal candidiasis was reported in an immunocompromised animal (*22*). Infection of the footpads with *C. albicans* has also been reported in an African pygmy hedgehog (*23*). The zoonotic potential of hedgehogs transmitting this infection to humans is therefore possible.

## Viruses

Several arboviral encephalitis viruses have been studied in the hedgehog. One study showed that hedgehogs are susceptible to even small amounts of Tahyna virus of the family *Bunyaviridae* and may produce a viremia level sufficient to ensure infection of mosquitoes (*24*). Hibernating hedgehogs may play a role as potential long-term reservoirs of the virus (*24*). Bhanja virus, also a member of the *Bunyaviridae*, has also been studied in European hedgehogs (*25*,*26*). Hedgehogs did not generate viremia titers, which indicates that the animals are likely not maintenance hosts for the virus (*25*). However, Hubalek et al. (*26*) suggested that the low level of antibodies may indicate that hedgehogs are passive distributors of infected ticks. The potential role of hedgehogs as natural hosts of these viruses and the risk that they may introduce these arboviruses in nondisease-endemic areas still need to be documented.

Tickborne encephalitis virus has been found in European hedgehogs (*27*). In at least 1 naturally occurring focus of the virus, the frequency of antibodies was considerably higher in hedgehogs than in small rodents, possibly related to the longer life cycle of hedgehogs as opposed to rodents, thus increasing the probability of reinfection (*27*). This study clearly demonstrated the importance of hedgehogs as hosts of ticks and reservoirs of the virus (*27*). Kozuch et al. (*28*) also demonstrated that alimentary infection of hedgehogs was possible. In addition, hedgehogs can maintain the virus during hibernation and may act as reservoirs during epidemic and interepidemic periods (*29*).

Crimean-Congo hemorrhagic fever (CCHF) is an important zoonotic disease of humans in the Middle East, as well as in Eastern Europe and Asia (*30*). The infection, which is often lethal, may be acquired through tick bite, contact with blood or tissues from infected livestock, or by treating CCHF-infected patients (medical personnel) (*30*). Studies in both European and African hedgehogs have demonstrated infection with CCHF virus (*31*,*32*). In European hedgehogs, viremia of sufficient intensity to infect feeding ticks does not develop (*33*). African pygmy hedgehogs appear susceptible to CCHF infection, but their role as amplifying hosts is yet undetermined; the South African hedgehog (*Atelerix frontalis*) does not appear to be an amplifying host (*32*). Although the response of individual species to CCHF virus varies markedly, hedgehogs act as important hosts to the immature stages of many of the tick species from which the virus has been isolated and therefore may be a potential source of infection (*32*).

Vizoso and Thomas (*33*) reported that a paramyxovirus of the Morbillivirus group had been isolated from 1 sick and several apparently healthy European hedgehogs. The sick animal displayed neurologic signs, ulcerated lesions on the soles of the feet, and histologic changes resembling canine distemper, another virus of the Morbillivirus group. Paramyxoviruses are commonly present in hedgehogs, and the possibility exists that the viruses cross species barriers, giving rise to infections in humans (*33*).

Herpesvirus infection has been reported in both European and African pygmy hedgehogs *34–36*). In both species, the primary site of infection has been the liver. In the first report, a feral European hedgehog had severe hepatic necrosis with extensive parenchymal hemorrhage and hyperemia (*34*). Herpesvirus particles were demonstrated by transmission electron microscopy. In a second report, involving a litter of orphaned European hedgehogs, 1 animal was necropsied and found to have a pale yellowish, friable liver with severely distorted general architecture (*35*). Culture of liver homogenate yielded herpeslike particles, which cause a cytopathic effect characteristic of alpha herpesvirus. A more recent report involved an African pygmy hedgehog owned by a private breeder (*36*). That animal was initially brought for treatment of acute posterior ataxia, which rapidly progressed to paresis. The hedgehog was treated for a prolapsed intervertebral disc and at first improved dramatically, but died 2 weeks after initial evaluation. On necropsy, the liver showed randomly distributed foci of necrosis and extensive inflammatory reaction. No bacteria were cultured, but viral culture produced an isolate that was subsequently identified as human herpes simplex virus. The source of the infection is uncertain, but the owner reported that family members occasionally suffered from cold sores (*36*).

A case of rabies in a hedgehog has been reported once (*37*). A family in inner-city Budapest found a hedgehog and, while playing with it, was exposed to typical salivary secretions. The animal later died and was confirmed to be positive for rabies. All family members were given postexposure prophylaxis to prevent infection.

Both European and African hedgehogs are susceptible to foot and mouth disease (FMD) virus. Although not technically a zoonosis, FMD could have a devastating impact on susceptible livestock. The earliest record of infection in hedgehogs suggested that the animals could have played a role in local spread of the disease (*38*). Typical vesicular lesions can develop on the hedgehog’s tongue, snout, and feet (*38*). Virus isolated from several hedgehogs trapped on or near infected premises during an outbreak of FMD in Britain was the same as that isolated from cattle in that outbreak (38*). The full cycle of infection between cattle and hedgehogs was also successfully demonstra*ted with African hedgehogs (*39*).

## Protozoa

Intestinal cryptosporidiosis was the cause of death in a captive juvenile African pygmy hedgehog (*40*). Large numbers of *Cryptosporidium* spp. developmental stages were present throughout much of the affected animal’s intestine. *Cryptosporidium parvum* is infectious to humans and represents a public health threat because of its waterborne transmission. The protozoan in this report was not identified to species, but zoonotic potential exists and appropriate precautions should be taken to prevent transmission to humans (*40*). Thorough hand washing with soap and water after handling hedgehogs is an important precaution to avoid contamination. Similarly, if one should clean up fecal materials from a pet, the use of disposable gloves and washing the hands afterwards is highly recommended.

## Conclusion

All pets have a flora of parasites and microbes, some of which are potentially zoonotic (*12*. Ownership of exotic pets may lead to increased exposure to various infectious agents, including exotic ones. However, the risk for exposure to zoonotic agents is substantially increased for people rescuing sick hedgehogs or rehabilitating them. Preventing illness depends on good hygiene and increased awareness, especially when young, elderly, or immunocompromised persons are involved (*12*). Pet hedgehog owners should systematically wash their hands after contact with their pets and make sure that any person who handled their pet will do so. Similarly, such pets should not be housed or handled in food preparation areas, as is recommended also for pet reptiles (*41*). Control of zoonotic diseases is dependent on breaking the cycle of transmission, and education is the key to control (*1*). Veterinarians should be the optimal source of providing correct information to clients making choices about owning exotic pets (*1*). Overall, ownership of exotic pets should not be encouraged because exotic animals and wildlife do not usually make good pets and can transmit zoonotic agents, as stated by the American Veterinary Medical Association and the National Association of State Public Health Veterinarians. In view of the popularity of hedgehogs and growing numbers of at-risk persons, veterinarians and other medical personnel must increase their understanding of the animal’s zoonotic potential.
